# Benchmarking surface tension measurement method using two oscillation modes in levitated liquid metals

**DOI:** 10.1038/s41526-021-00137-9

**Published:** 2021-03-09

**Authors:** Nevin Brosius, Kevin Ward, Evan Wilson, Zachary Karpinsky, Michael SanSoucie, Takehiko Ishikawa, Satoshi Matsumoto, Ranga Narayanan

**Affiliations:** 1 University of Florida Department of Chemical Engineering, Gainesville, FL USA; 2 NASA Marshall Space Flight Center, Huntsville, AL USA; 3 Human Spaceflight Technology Directorate, Japan Aerospace Exploration Agency, Tsukuba, Ibaraki Japan

**Keywords:** Techniques and instrumentation, Fluid dynamics, Chemical engineering, Materials science

## Abstract

The Faraday forcing method in levitated liquid droplets has recently been introduced as a method for measuring surface tension using resonance. By subjecting an electrostatically levitated liquid metal droplet to a continuous, oscillatory, electric field, at a frequency nearing that of the droplet’s first principal mode of oscillation (known as mode 2), the method was previously shown to determine surface tension of materials that would be particularly difficult to process by other means, e.g., liquid metals and alloys. It also offers distinct advantages in future work involving high viscosity samples because of the continuous forcing approach. This work presents (1) a benchmarking experimental method to measure surface tension by excitation of the second principal mode of oscillation (known as mode 3) in a levitated liquid droplet and (2) a more rigorous quantification of droplet excitation using a projection method. Surface tension measurements compare favorably to literature values for Zirconium, Inconel 625, and Rhodium, using both modes 2 and 3. Thus, this new method serves as a credible, self-consistent benchmarking technique for the measurement of surface tension.

## Introduction

The accurate measurement of thermophysical properties is imperative for the future of countless areas of manufacturing^1^ and extends its importance into space exploration with the recent push for the Artemis program, in-space manufacturing, and in-situ resource utilization^2^. The reliability of processes like crystal growth, additive manufacturing, and welding depend on precise knowledge of thermophysical phenomena^3,4^. However, thermophysical property measurement of high-temperature materials like liquid metals, glasses, and oxides is difficult using conventional means because of high surface reactivity^5^. The advent of containerless material processing using aerodynamic^6^, acoustic^7,8^, electromagnetic^9^, and electrostatic^10,11^ levitation technologies has provided an avenue for further improvements in the accuracy of thermophysical property measurements and can also be used to provide insights into other important material behavior like phase equilibria and solidification dynamics^9,10^. Surface tension, an important thermophysical property, can be measured using levitation technologies by exploiting the natural frequency *f*_*n*_ at which a spherical liquid droplet comes to rest, given by^12^

1 $${f}_{n}=\sqrt{\frac{n(n-1)(n+2)\gamma }{3\pi M}}$$

where *γ* is the surface tension, *n* is the normal mode of oscillation, and *M* is the mass. By experimentally finding the natural frequency *f*_*n*_, one can solve Eq. (1) for surface tension.

Electrostatic levitation holds several advantages to other levitation techniques. Aerodynamic and acoustic levitation both require the presence of a fluid medium, which can present engineering challenges like contamination, viscous effects^5^, and large deviations in the spherical base state^13^. Electromagnetic levitation experiments take place in vacuum but also suffer from the issue of asphericity^14^. The fundamental way in which a droplet is magnetically levitated produces eddy formation within the liquid droplet, the flows of which can highly disturb the droplet’s natural shape due to pressure gradients. The non-spherical nature of the droplet when magnetically levitated causes its frequency response to split degenerate *n* = 2 modes into separate frequencies. However, in electrostatic levitation, the only requirement for levitation is to maintain high enough surface charge to balance the weight of the droplet^11^, which does not cause strong internal flows and leads to a more spherical droplet. Experimental observations^5^ have also noted a more spherical droplet, which leads to a single frequency response. Faraday forcing, as explained in the following section, uses electrostatic levitation as a means to measure surface tension.

### Faraday forcing

**Fig. 1:**
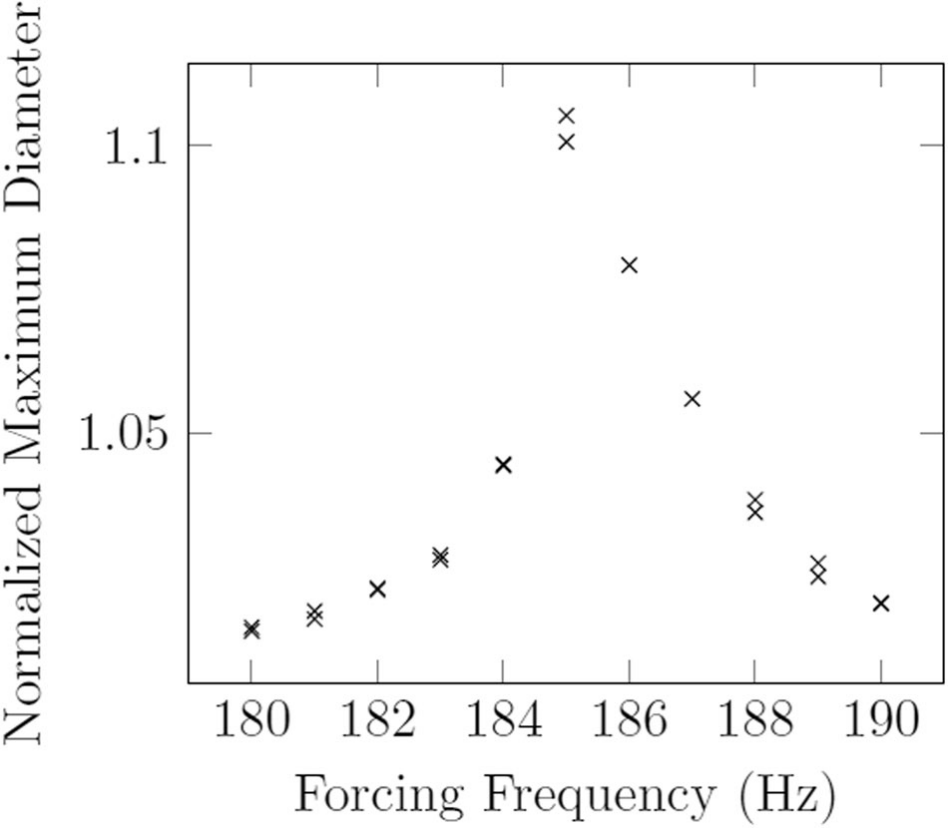
The Faraday forcing method using the normalized maximum diameter of the oscillating sample (from Brosius et al.^15^). Sample is 35.027 mg Zirconium at 1800 ^∘^C.

Introduced in this work is a refinement of the Faraday forcing method described in 2018 by Brosius et al.^15^ to include the mode *n* = 3 in addition to a modified resonance quantification method. The mode *n* = 3 has been previously observed in electrostatic levitation systems using relatively large (*D* > 4 mm) droplets in the investigation of the stability of levitated Zirconium as a function of size and temperature^16^. The current work aims to include the resonance quantification of mode *n* = 2 and mode *n* = 3 as a benchmarking method for determining surface tension of a material using just one levitated sample. The Faraday forcing method was introduced as a method to stimulate resonance in electrostatically levitated droplets using a frequency sweep, where the droplet is forced with an incrementally changing frequency in a range surrounding the predicted natural frequency. The droplet’s deformation is recorded as it is forced. Resonance (increasing amplitude of droplet deformation) occurs as the forcing frequency approaches that of the droplet’s natural frequency. In the earlier work, the magnitude of this deformation (i.e., the magnitude of resonance) was quantified by the normalized maximum diameter, defined as the maximum prolate diameter in resonance divided by the resting diameter of the droplet. This was due to the fact that only the first principal mode was sought, which corresponds to a prolate–oblate deformation. It is important to note that the normalized maximum diameter is limited to mode *n* = 2 deformations; an alternative metric presented herein is required to extend this to higher order modes. In Fig. 1 we observe the quantitative representation of a frequency sweep, known as a resonance curve. The forcing frequency corresponding to the maximum of this curve is termed the resonant forcing frequency and is substituted into Eq. (1) to solve for surface tension.

**Fig. 2:**
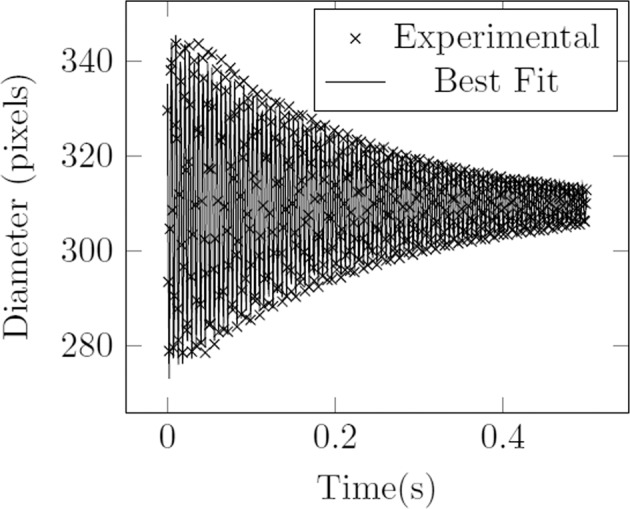
A typical set of data from the pulse decay technique and its fit to an exponentially decaying sinuosoidal function. Sample is 35.027 mg Zirconium at 1800 ^∘^C. cf^15^.

The method stands in contrast to the so-called pulse-decay technique^5^, where a droplet is forced to oscillate, and the forcing is abruptly stopped. The deformation of the droplet returning to rest is recorded as a function of time and fitted to an exponentially decaying sinusoidal function, as seen in Fig. 2. The frequency at which the oscillation decays approaches the fundamental frequency, corresponding to *n* = 2, of the droplet. This pulse-decay technique is the current industry standard and has the advantage of being able to measure both surface tension and viscosity. The Faraday forcing method is thought to avoid some of the control system disturbances that may arise with the pulsed perturbation and may provide improved accuracy in surface tension measurement. The two techniques are compared in Table 1.

**Table 1:** Comparison of surface tension results using the Faraday forcing method from Brosius et al.^15^ and existing literature values using the pulse-decay technique.

Material	Temp. (^∘^C)	Measured surface tension (Nm^−1^)	Literature surface tension (Nm^−1^)	% Difference
*Zirconium*	1700	1.48	1.52^27^	−3%
*Zirconium*	1800	1.41	1.51^27^	−6%
*Ti*_39.5_*Zr*_39.5_*Ni*_21_	950	1.53	1.670^28^	−14%

Errors in measurement stemming from the deformation amplitude, electric field strength, gravity, and frequency step size (precision of the frequency sweep) range from 1–5%.

By expanding the Faraday forcing method to include the characterization of droplet resonance in mode 3, this method can serve as a self-consistent scheme for the measurement of surface tension. In employing this method, one performs a frequency sweep around the expected fundamental (*n* = 2) mode frequency and records the result. The sample is then subsequently subject to a second frequency sweep around the expected *n* = 3 frequency. The frequency at which *n* = 3 is expected to resonate most strongly is also predicted by Eq. (1). Resonance is quantified with the projection of the Legendre polynomials used to characterize the shape of the droplet’s outline and provides a distinct modification to the method of Faraday forcing for the measurement of surface tension.

## Results and discussion

Each frequency sweep, whether for mode 2 or mode 3, consists of a small, constant forcing amplitude and an incrementally changing forcing frequency which is superimposed over the control voltage of the upper and lower electrodes. Images are captured using a high-speed camera and analyzed to find the amplitude of the projection of each principal mode of oscillation as a function of time for each forcing frequency of the sweep. The difference in the magnitude of resonance can be qualitatively observed in both modes 2 and 3 for a sample of Inconel 625 in the linked video. The forcing frequency corresponding to the sample’s maximum deviation is termed the resonant forcing frequency and is inserted into Eq. (1) to solve for surface tension. Table 2 summarizes the measurements obtained during this work. The data from Brosius et al. was also reanalyzed using the projection method, which is elaborated upon in the following sections. Surface tension measurement errors are determined by the small, yet finite deformation amplitude, the frequency sweep precision, and gravity/electric field effects and range from 1 to 5%.

**Table 2:** Table of results using the Faraday forcing method for modes 2 and 3.

Material	Temp (^∘^C)	Literature ST (Nm^−1^)	Mode 2 Trials	Mode 3 Trials	Mode 2 ST (Nm^−1^)	Mode 2% difference	Mode 3 ST (Nm^−1^)	Mode 3% difference
*Zirconium*	1800	1.506^29^	14	12	1.38 ± 0.04	−8%	1.39 ± 0.021	−8%
*Inconel 625*	1350	1.7^30^^a^	11	4	1.72 ± 0.15	1%	1.75 ± 0.041	3%
*Rhodium*	1800	2.0291^31^	3	1	1.96 ± 0.01	−4%	1.96 ± 0.003	−3%

% difference corresponds to the difference between the measured surface tension value and the accepted literature value for modes 2 and 3, respectively.

^a^There is no existing literature value to the authors’ knowledge, so the value for Inconel 600 is used as a reasonable comparison.

### Measurement of surface tension

The primary impact of this work is a proof-of-concept for a benchmarked measurement of surface tension using resonance of multiple modes of oscillation in levitated liquid materials. In the earlier work of Brosius et al.^15^, it was shown that the continuous Faraday forcing method yielded consistent results that agreed reasonably well with literature values (from the pulse-decay method). It was hypothesized in that work that the incorporation of higher-order modes that come from the pulse could cause a shift in natural frequency because of nonlinear interaction between modes. In Table 2, the values for modes 2 and 3 are remarkably consistent for samples of Zirconium, Rhodium, and Inconel 625, certifying the accuracy of the continuous forcing technique. The literature values for these three materials are also all within 10% of this report’s measurements (note that literature values for Inconel 625 were unavailable and the value for Inconel 600 was used).

### Comparisons with the prior quantification of resonance

**Fig. 3:**
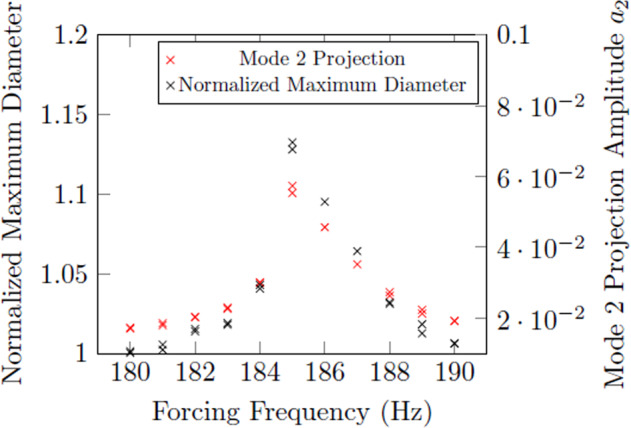
Comparison of projection and normalized diameter results for the Faraday forcing method on mode 2 resonance. Sample is 35.027 mg Zirconium at 1800 ^∘^*C*.

In the previous work, the magnitude of resonance was quantified in mode 2 by measuring the normalized maximum diameter of the droplet in resonance. This method, known as the diameter method, was straightforward because the droplet’s shadow oscillates between an oblate and prolate ellipse in mode 2. An important update in the current work is the modified approach to quantifying the resonance of the droplet using the projections of Legendre polynomials (stemming from a projection of the spherical harmonics). While the prior diameter-based method was more straightforward, it did not lend the generality that the projection method provides – that is, the current method can quantify any modal deformation (provided the droplet is adequately spherical when unforced and perturbations are axisymmetric). As might be expected, the two methods do agree in the characterization for mode 2 resonance, shown in Fig. 3.

### Comparisons with indirect methods of resonance quantification

**Fig. 4:**
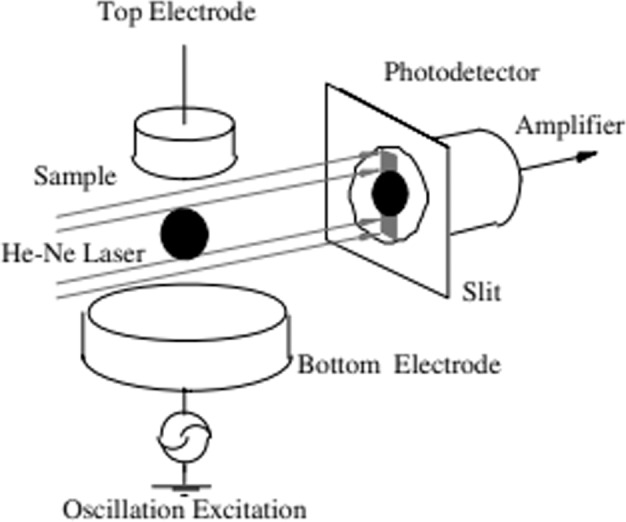
Area array setup at JAXA used to characterize the behavior of the oscillating droplet. A He-Ne laser is projected onto the levitated droplet and the change in light intensity received by the photodetector is proportional to the droplet’s shadow area (Borrowed from^17^ with permission from *Elsevier, Inc*).

The magnitude of resonance may also be characterized using indirect (non-image-based) methods. For example, the electrostatic levitator at the Japan Aerospace and Exploration Agency (JAXA) in Tsukuba, Japan uses a He-Ne laser to cast a shadow on a photodetector, which is blocked by the sample, as shown in Fig. 4^17^. This setup is known as an area array and is also used to characterize droplet behavior on the Electrostatic Levitation Furnace (ELF) on the International Space Station, also operated by JAXA. The output signal from the receiver is directly proportional to the shadow area and provides a distinct advantage in computation time when compared to imaging-based methods. The strength of oscillations in the droplet’s area is proportional to the magnitude in which the droplet is oscillating in mode 2. The definition below is used to quantify the droplet shadow’s area deviation given time-dependent data.

2 $${A}_{{\mathrm{meansq}}}=\frac{1}{N}\mathop{\sum }\limits_{i=1}^{N}\left(\right.\frac{{A}_{i}-{A}_{{\mathrm{avg}}}}{{A}_{{\mathrm{avg}}}}{\left)\right.}^{2}$$

where *A*_avg_ is the time-averaged shadow area of all instantaneous shadow area values *A*_*i*_.

**Fig. 5:**
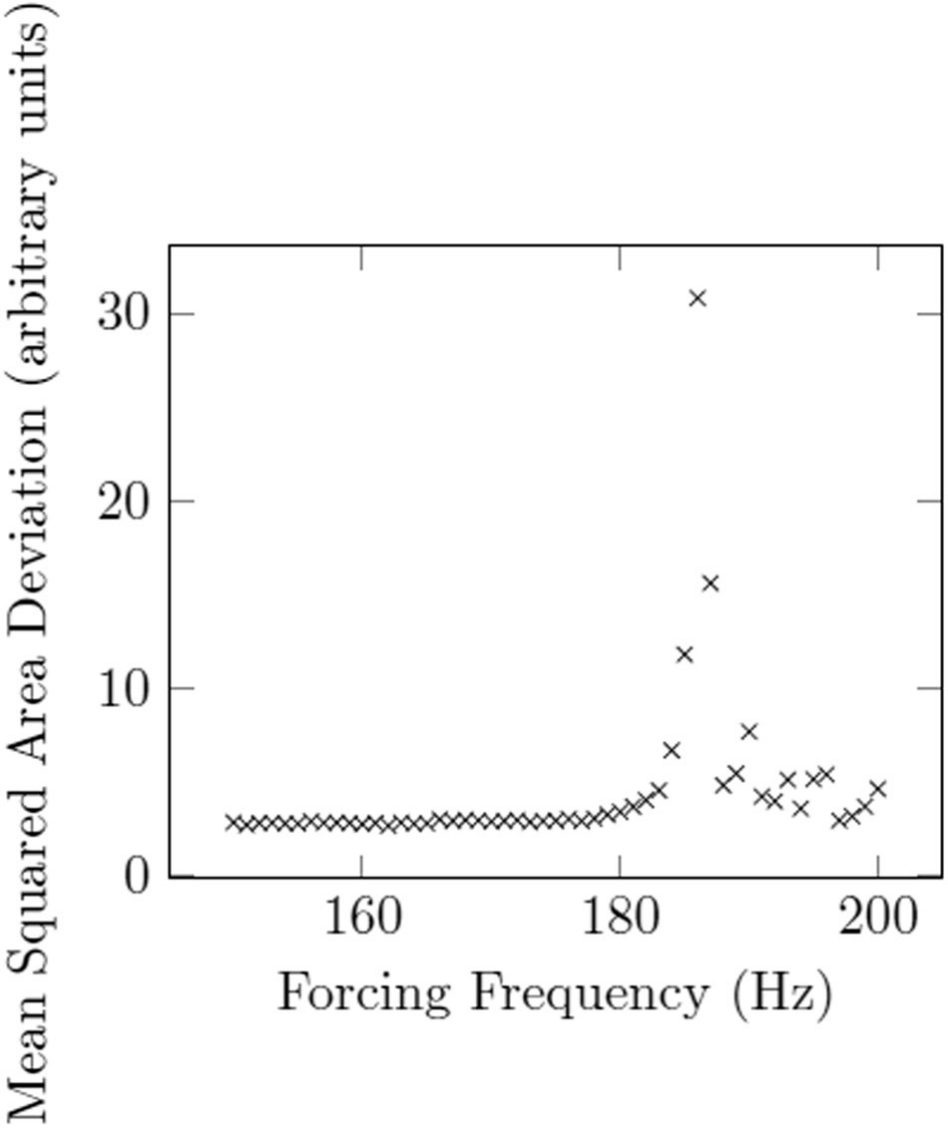
Resonance curve of Rhodium (45 mg) oscillating in mode *n* = 2, obtained using an area array at Japan Aerospace Exploration Agency (JAXA). The mean squared area deviation of the droplet’s shadow over time corresponds to the magnitude of resonance. The resonant frequency is the frequency corresponding to the maximum of this curve.

**Fig. 6:**
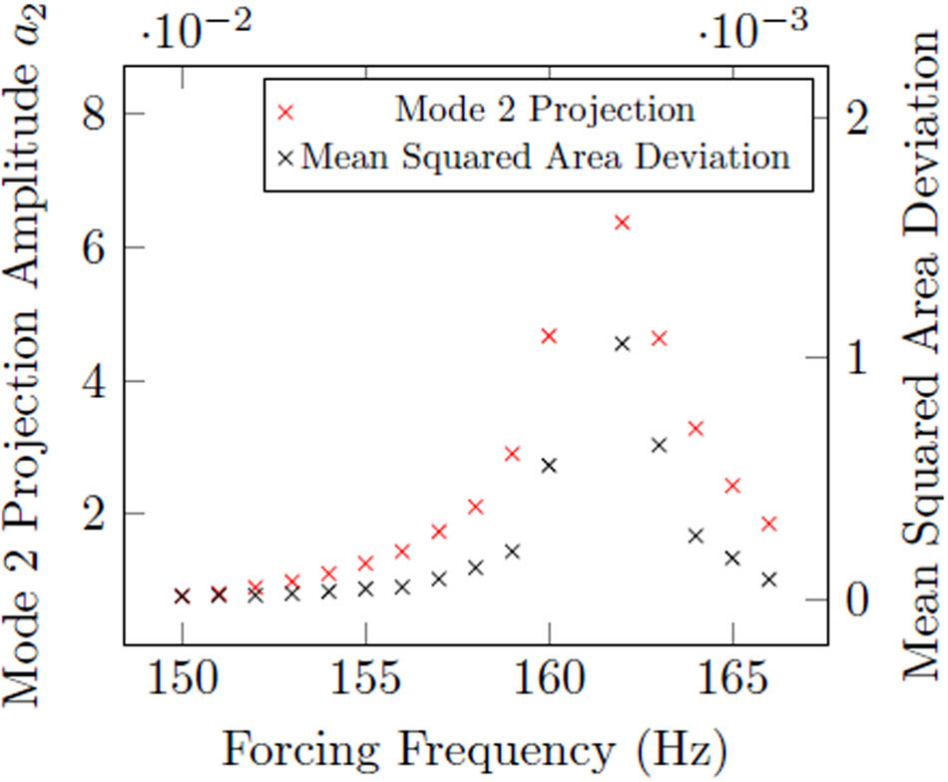
Comparison of results from an area array and projection method for mode 2 resonance. Sample is 57.345 mg Inconel 625 at 1350 ^∘^*C*.

As shown in Fig. 5, the area array can be used to confirm the resonance of mode 2 for surface tension measurement. For comparison, Fig. 6 shows artificially created array data from video data obtained at NASA MSFC’s ESL Laboratory and compared to the results obtained using projections in mode 2. One observes that the forcing frequency resulting in the maximum projection amplitude coincides with the forcing frequency obtained in quantifying the droplet’s oscillation using the shadow area.

While indirect area arrays may be used to effectively quantify the droplet’s deformation in mode 2, the method is unsuccessful in quantifying resonance of mode 3. The reasoning for this discrepancy lies in the fact that the area of the shadow is negligibly affected by deformation in mode 3. This is proven using an approach akin to that of the application of the Rayleigh work principle in liquid bridge stability^18^. The shadow area *A* of the deformed droplet is given by the following integral:

3 $$A={\int\nolimits_{0}^{2\pi }}\frac{{(R+\delta r(\theta ))}^{2}}{2}d\theta$$

where *δr*(*θ*) is the droplet shadow’s deviation from a circle and *R* is not the physical resting radius *R*_0_ but rather a characteristic radius defined such that the volume *V* is constant, that is:

4 $$V=\frac{4}{3}\pi {R}_{0}^{3}=2\pi {\int\nolimits_{0}^{\pi }}\frac{{(R+\delta r(\theta ))}^{3}}{3}\sin (\theta )d\theta$$

The mode 2 and mode 3 disturbances are given by the Legendre polynomials for *n* = 2 and *n* = 3, respectively. That is,

5 $$\delta r(\theta )=\mathop{\sum }\limits_{n=2}^{3}{a}_{n}{P}_{n}(\cos (\theta ))$$

6 $${P}_{2}(\cos (\theta ))=\frac{1}{2}(-1+3{\cos }^{2}(\theta ))$$

7 $${P}_{3}(\cos (\theta ))=\frac{1}{2}(-3\cos (\theta )+5{\cos }^{3}(\theta ))$$

**Fig. 7:**
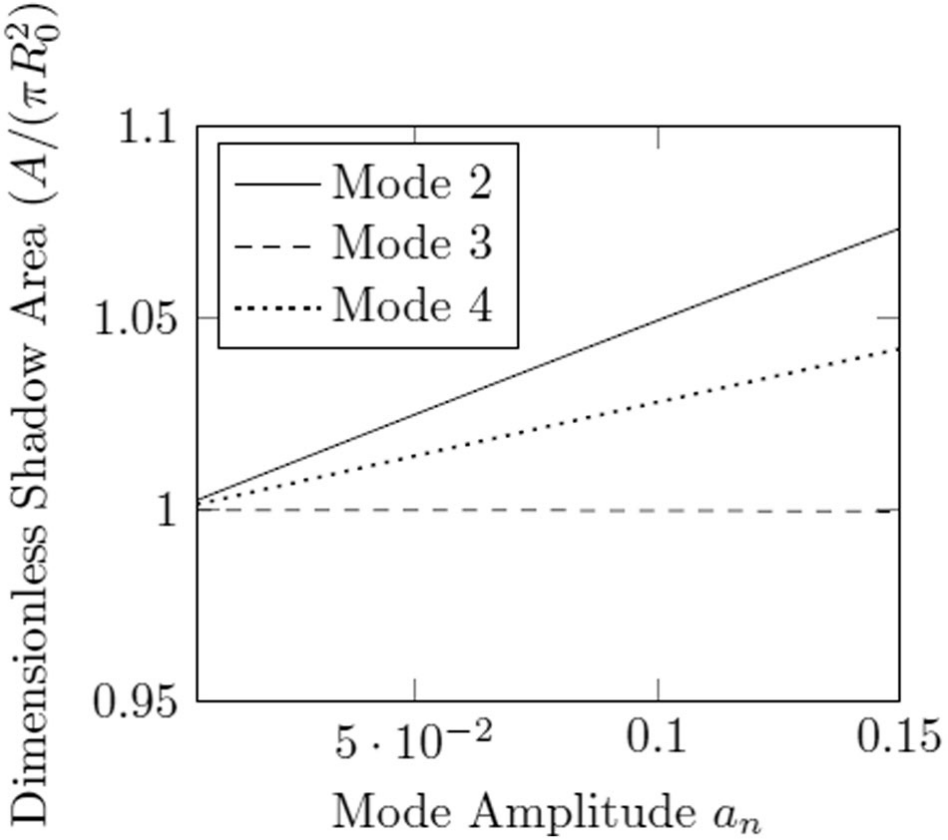
Comparison of shadow area change for modes 2, 3, and 4 as a function of deformation amplitude. The dimensionless shadow areas ($A/(\pi {R}_{0}^{2})$) are calculated using the analytical spherical harmonics of the droplet with superimposed mode 2, mode 3, mode 4 functions at varying amplitudes *a*_*n*_ with volume constrained to $\frac{4}{3}\pi {R}_{0}^{3}$.

The area of the droplet’s shadow may then be found analytically as a function of the amplitude *a*_*n*_ of each normal mode. Shown in Fig. 7 is the relationship between the shadow area (normalized with respect to the resting area, $\pi {R}_{0}^{2}$) and the amplitude, *a*_*n*_. This indicates that, although the droplet may be oscillating strongly, a signal reflecting the area of the droplet’s shadow is insufficient in characterizing the resonance of mode 3. However, the mode *n* = 4 is included to illustrate that this area array setup can still be used in conjunction with the continuous Faraday forcing method with higher harmonics, provided that *n* is even.

**Fig. 8:**
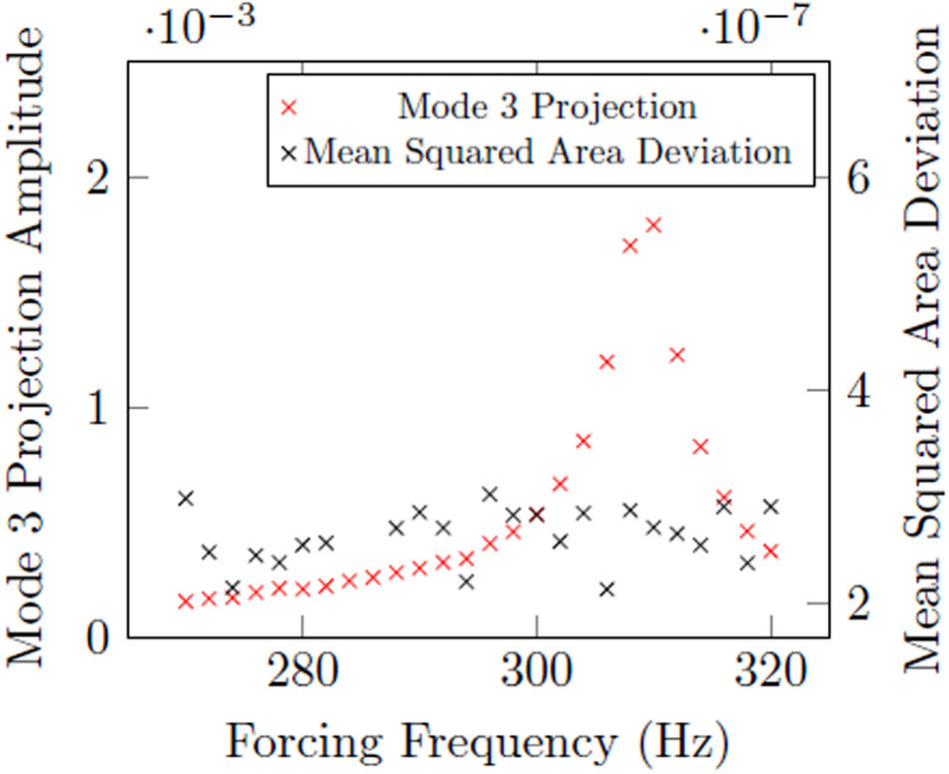
Comparison of results from an area array and projection method for mode 3 resonance. Sample is 57.345 mg Inconel 625 at 1350 ^∘^C.

This conclusion is further confirmed in analyzing experimental data where mode 3 was definitively observed, by attempting to quantify resonance using an artificial area array. Shown in Fig. 8 is a mode 3 frequency sweep using Inconel 625 where the projection method yields a strong resonance curve with a clear resonant frequency, while the mean squared area deviation does not yield any conclusive result.

### Sources of error

#### Deformation amplitude

It is well-understood that the oscillation frequency of a droplet is affected by its displacement amplitude *a*_*n*_ (referred to as *ϵ* in Tsamopolous & Brown^19^). It may be thought that since the method is reliant on the determination of the most resonant point in a frequency sweep, the frequency at which the droplet most strongly deforms must be slightly shifted by a factor *δf*/*f* (given by Eq. (8)) from its true natural frequency given by Eq. (1) and decrease the accuracy of the surface tension measurement.

8 $$\frac{\delta f}{f}=\frac{1}{2}(1.17037){a}_{2}^{2}$$

While this is a valid concern in principle, all experiments conducted in this work exhibited deformation amplitudes which would correspond to a maximum frequency shift given by ref. ^19^ of less than 2% and thus within the stated error of 1–5% in the measurement of surface tension. The maximum deviation observed in this work was *a*_2_ = 0.1, corresponding to *δf*/*f* = 0.008 and an estimated error of 1.6%.

#### Electric and gravitational field effects

The effect of electric field and gravitational "sagging" has been quantified by several theoretical works, involving multiple parameter perturbation methods. Both the strength of the electric and gravitational fields seem to shift the natural oscillation frequency of the droplet downwards and attribute error akin to that of non-negligible amplitude displacements. Theoretical work by Feng et al.^20^ and experimental work by Rhim et al.^11^ established a relationship between the frequency shift and a correction factor which depended on the charge, electric field, and the shape of the droplet’s deformation.

9 $${\omega }_{l}^{* }={\omega }_{l}[1-({A}_{0}^{\,{<}\,2,1 \,{>}\,}(l)/4{\omega }_{l}^{2}){\epsilon }_{1}^{2}]$$

where *ω*_*l*_ is the natural angular frequency given by Eq. (1), ${A}_{0}^{\,{<}\,2,1 \,{>}\,}(l)/4{\omega }_{l}^{2}$ is a correction factor, and *ϵ*_1_ is the dimensionless electric field strength.

For all samples in this work, this error is estimated to be as low as 0.5% (in small samples) but can be as high as 1.5%. Analogous to the effect of amplitude on the natural oscillation frequency, the shift in mode 3 oscillation from the electric and gravitational fields is much smaller than the shift in mode 2 oscillation.

The error stemming from electric and gravitational fields is one of the strong incentives to levitation processing in microgravity. Because of the lack of gravity, the equilibrium shape of a levitated droplet is more perfectly spherical and also requires a significantly weaker electric field to maintain its levitation.

#### Precision of the frequency sweep

The final significant source of error involved in this procedure’s measurement of surface tension involves the frequency step size in the sweep. All frequency step sizes in this work range from 0.5–1.0% of the natural frequency *f*_*n*_ and thus the error can be estimated as 1–2%. However this error can be reduced to arbitrary precision by lowering the step size of the frequency sweep.

### Experimental challenges

There are several challenges that encompass the practice of this method which either directly or indirectly stem from the presence of gravity. The levitation process on Earth can only successfully levitate samples which have suitable thermophysical properties. For example, very dense materials like Platinum or Iridium are difficult to levitate because of high density and high melting point. The charge of the droplet, needed for levitation control, changes as the sample is heated due to thermionic emission. In some cases, this can cause dramatic changes in surface charge and result in the loss of the sample. Materials that cannot be subcooled sufficiently below melting point (or otherwise have high vapor pressures) present an additional challenge with constant evaporation. This evaporation limits processing time and can cause potential fouling of the sensitive equipment within the chamber. Evaporation can be limited by levitating samples within a pressurized atmosphere, but this is challenging on Earth for liquid metals and alloys because the strength of electric field required for levitation is beyond the threshold for electrical breakdown and results in arcing. There are also challenges that result in the nonlinear dynamics of the spherical droplet being subject to an oscillatory forcing. As described in Methods, the droplet is assumed to be *ϕ*-symmetric. However, this is an assumption that is based on experimental observation and is not strictly obeyed. The degenerate modes (say *m* = −2, −1, 0, 1, 2 for *n* = 2) all ordinarily coexist at the same frequency and all have some probability of being excited. However, in this experiment, the *m* = 0 mode is most often observed. When one of the degenerate modes are excited, this causes an "off-axis” oscillation and torque from the electric field can cause substantial spinning of the droplet. This spinning splits the degenerate modes into three frequencies. Spinning can also be stimulated without deformation of the droplet due to photon pressure of the heating laser. These dynamics can be minimized by lowering the amplitude of forcing and allowing the droplet to fully relax in between forcing frequencies. To accommodate these challenges, the frequency sweeps garnering highly nonlinear coupling behavior are analyzed but not used in the measurement of surface tension because of the shifting of the natural frequencies.

### Conclusions and future work

This work confirms that the principle behind using a series of frequency sweeps to identify multiple modes of oscillation in levitated liquid droplets can serve as a self-consistent, benchmarking method for the measurement of surface tension. Three materials – Zirconium, Rhodium, and Inconel 625 – have exhibited consistent, predictable behavior in the resonance of the first two principal modes of oscillation which serves to confirm the surface tension measurement in a fashion superior to repetition alone.

Visualization methods affect the ability to observe odd harmonics of oscillation, as shown in Fig. 8, if using an indirect visualization setup like an area array. However, as indicated in Fig. 7, higher harmonics corresponding to even values of *n* may be sensible in future experiments using area arrays. The difficulty in experimentally attaining higher harmonics lies in the higher energy states and correspondingly high damping rates, which are limited by the forcing voltage and the charge of the droplet. This difficulty may be alleviated by operating on samples in low gravity with low fundamental frequencies given by Eq. (1) (i.e., low surface tension and large mass).

The method of Faraday forcing for the measurement of surface tension could be extended to resonance of higher order modes and an expanded array of materials such as glasses, oxides, and alloys. Levitation experiments on JAXA ELF which use an area array for sample monitoring can be effectively utilized in observing further benchmarking of the method using even harmonics. Space experiments can also be used to measure the surface tension of materials of high density such as Gold and Platinum which are difficult to electrostatically levitate on Earth.

## Methods

### Experimental methods

All experiments employing the self-consistent measurement method were conducted at the Electrostatic Levitation (ESL) Laboratory at NASA Marshall Space Flight Center (MSFC). Experiments performed at Japan Aerospace Exploration Agency for reasons of comparisons with the aforementioned method are explained in the Discussion. The process of levitating the sample is identical to the methods outlined in Brosius et al.^15^. Samples are levitated as a solid using an electrostatic position feedback system, which controls both vertical and horizontal positioning. The levitation chamber is first evacuated using a turbomolecular pump to 10^−7^ torr. Electric charge is applied to the sample with a UV beam and replenished, if needed, throughout processing. The sample is melted using a fiber optic laser and superheated approximately 50 ^∘^C above melting point and allowed to radiatively cool to solidification to record density measurements. A single wavelength pyrometer (Advanced Energy IMPAC IGA 140) is used to measure the temperature within the range 300 to 3000 ^∘^C. In surface tension measurements, the sample is melted and then subcooled below the melting point (if possible) to minimize evaporation. Upon reaching the desired temperature, the laser is used to maintain constant temperature. A waveform generator and amplifier are connected to the upper electrode to allow the user to impose an oscillatory electric field. Droplet behavior is characterized with a high-speed camera at 5000fps and 512 by 512 pixels, typically for durations of 1–1.5 s. The sample is weighed both before and after processing to quantify evaporation.

The natural frequencies for both modes 2 and 3 are initially predicted using the droplet’s mass and accepted literature surface tension value with equation 1. Modes 2 and 3 are both discovered using a method known as a frequency sweep, following the precise procedure of Brosius et al. For each frequency of a frequency sweep, an oscillatory voltage (known as a forcing frequency) is applied to the top/bottom electrodes for 1–2 s. Upon completion of each forcing frequency, the oscillatory voltage is turned off and the droplet’s deformations can come to rest before starting the next forcing frequency (typically 1 s). The sweep is centered about the predicted natural frequency and, depending on confidence level, the step size (difference between one forcing frequency and the next) is 0.5–5 Hz, with 20–30 total frequencies tested. For example, the mode 2 natural frequency of a 35.027 mg Zirconium sample at 1800 ^∘^C is predicted to be 185 Hz, so the sweep would encompass frequencies ranging between 170–200 Hz, taking steps of 1 Hz. After finishing the mode 2 frequency sweep, the droplet remains levitated but is solidified to suppress evaporation. The videos from the high-speed camera are previewed after the sweep prior to download and resonance is characterized by eye before beginning the mode 3 sweep.

If there is no published data for the material of interest, multiple frequency sweeps are performed to find the resonance point of mode 2. First, a broad (100–200 Hz) sweep of large step size is used to force the droplet to resonate in mode 2. Subsequent sweeps follow with narrowing frequency range and decreasing step size until desired accuracy is achieved.

The fashion in which the mode 3 sweep is conducted depends on the preliminary results from the mode 2 sweep. If there is conclusive video evidence that the mode 2 sweep yielded strong resonance at a certain frequency (known as *f*_max_), the mode 3 sweep will be centered on its corresponding mode 3 frequency. This corresponding mode 3 frequency, given by Eq. (1), is $\sqrt{30/8}{f}_{max}$. The value of *f*_max_ may be different than the predicted natural frequency from literature values. If there is not conclusive video evidence of resonance, the frequency sweep for mode 3 is chosen in accordance to accepted literature values, if they exist. If there are no published data for the material and no conclusive video of mode 2 is observed, mode 3 will be sought using a progressively narrowing set of frequency sweeps, identical to the fashion in which mode 2 is sought. The sample is remelted, subcooled (if possible), and the mode 3 frequency sweep is conducted, taking steps of 0.5–5 Hz.

All videos are processed and subsequently analyzed using ImageJ to obtain the droplet shadow area and outline as a function of time. Mathematica is used in the batch processing of the projection method and evaporation quantification. The forcing frequency which yields the highest magnitude of resonance for a given frequency sweep is termed the resonant frequency and plugged into Eq. (1) to calculate surface tension.

### Analysis method

The method in which resonance is quantified is termed herein as the projection method, where Legendre polynomials corresponding to each mode’s precise shape (as defined by Rayleigh^12^ and often employed as a means of quantifying spherical deformations using image analysis^21^) are projected on to the outline of the droplet. While this method returns the same results for mode 2 as the method outlined in Brosius et al., it is more consistently used for both modes 2 and 3 and thus serves as a more robust quantification of resonance, which can be used for any axisymmetric deformation (see the Discussion for a detailed comparison of the projection method to the diameter method). In the absence of external forces and in the limit of low viscosity^22–24^, deformations on a spherical surface can be described by^25^

10 $$r(\theta ,\phi ,t)={R}_{0}+\mathop{\sum }\limits_{n=2}^{\infty }\mathop{\sum }\limits_{m=-n}^{n}{A}_{n}^{m}{e}^{im\theta }{P}_{n}^{m}(\cos (\theta ))\cos (2\pi {f}_{n}t)$$

where *R*_0_ is the resting radius of the sphere, ${A}_{n}^{m}$ are deformation amplitudes, ${P}_{n}^{m}$ are the associated Legendre polynomials, *n* is a normal mode of oscillation, *m* represents degenerate modes of *n*, and *f*_*n*_ is given by Eq. (1).

Experimental findings by Rhim et al.^5^ noted that a single axisymmetric mode (*m* = 0 in Eq. (10)) is preferentially excited compared to its non-axisymmetric counterparts (*m* = ±1, ±2) when electrostatically levitated, meaning the droplet could be well-approximated by a simplified version of Eq. (10)

11 $$r(\theta ,t)={R}_{0}+\mathop{\sum }\limits_{n=2}^{\infty }{A}_{n}{P}_{n}(\cos (\theta ))\cos (2\pi {f}_{n}t)$$

where *P*_*n*_ are the Legendre polynomials and *f*_*n*_ remains the same. Charge and gravitational effects unique to electrostatic levitation are derived by Rayleigh^26^. In this work, these effects amount to a correction factor^20^ to the natural frequency given by Eq. (1) further explained in the Discussion.

Starting with an outline of the droplet, which is found through image analysis as a function *r*(*θ*, *t*), the function *δr*(*θ*, *t*) is defined as the normalized difference between the outline and the resting radius of the droplet.

12 $$\delta r(\theta ,t)=\frac{r(\theta ,t)}{{R}_{0}}-1=\mathop{\sum }\limits_{n=2}^{\infty }{a}_{n}{P}_{n}(\cos (\theta ))\cos (2\pi {f}_{n}t)$$

Note that *a*_*n*_ are now normalized with respect to the resting droplet’s radius, that is *a*_*n*_ = *A*_*n*_/*R*_0_. Since the Legendre polynomials obey the relationship:

13 $${\int\nolimits_{0}^{2\pi}}{P}_{n}(\cos (\theta )){P}_{m}(\cos (\theta ))d\theta =\frac{2}{2n+1}{\delta }_{mn}$$

one may find the temporal amplitudes, *α*_*n*_(*t*), by way of projection, that is

14 $${\alpha }_{n}(t)={a}_{n}\cos (2\pi {f}_{n}t)=\frac{2n+1}{2}{\int\nolimits_{0}^{2\pi}}{P}_{n}(\cos (\theta ))\delta r(\theta ,t)d\theta$$

**Fig. 9:**
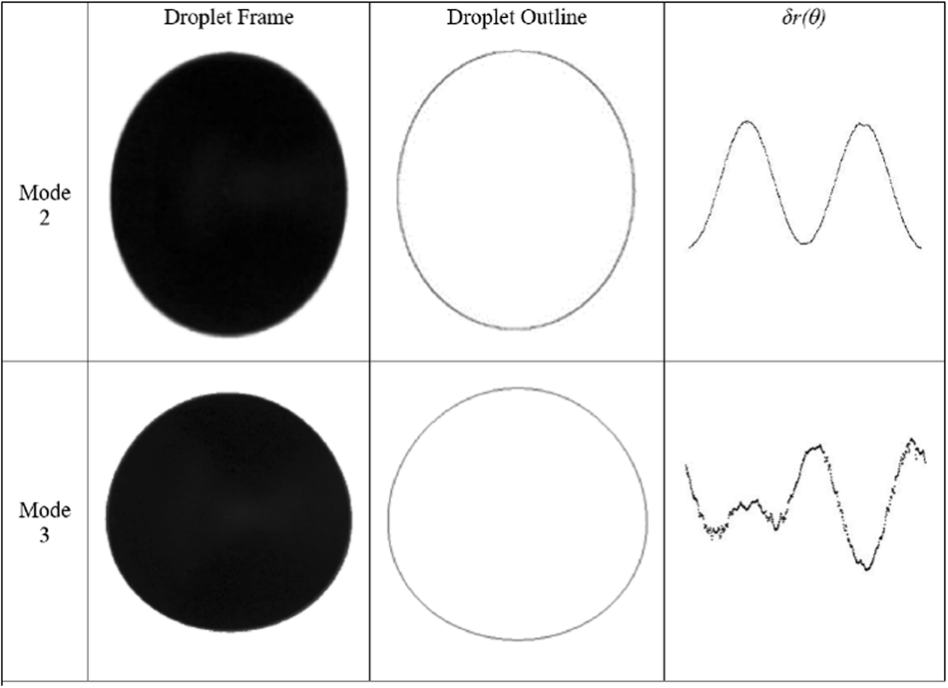
A graphical illustration of the image analysis process for the projection method on mode 2 (top) and mode 3 (bottom). For each video frame, ImageJ software is used to convert the droplet’s shadow to an outline and subsequently convert the outline to polar coordinates. These polar coordinates are then projected onto the corresponding Legendre polynomial over the domain [0,2*π*] to find the projection amplitudes, *a*_*n*_.

**Fig. 10:**
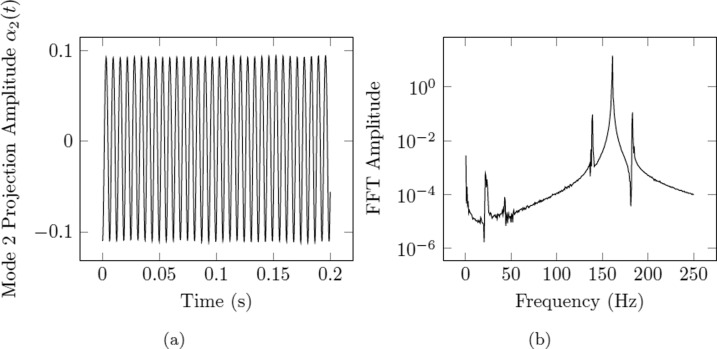
Dynamic output of the projection method for a droplet subjected to modal oscillations. **a** The dynamic projection amplitude, *α*_2_(*t*) as a function of time, is computed for each forcing frequency after completing the image analysis procedure described in Fig. 9. **b** Logarithmic representation of the FFT amplitudes of the dynamic projection amplitude. The maximum peak corresponds to the dominant frequency of oscillation in the droplet.

Using this definition of the projection amplitudes, one can therefore use the deformation outline *δr* as obtained through image processing and perform a numerical integration scheme to quantify the amplitude of a given mode. The process is given graphically in Fig. 9. Performing this calculation for each frame of the video returns a graph like that shown in Fig. 10a for each forcing frequency.

Assuming sinusoidal behavior with total acquisition time *T*, one may assume the following relationship between the constant *a*_*n*_ and the time-dependent data given by *α*_*n*_(*t*) when the number of oscillation cycles is large:

15 $${a}_{n}=\sqrt{2}{\left(\frac{\mathop{\int}\nolimits_{0}^{T}{({\alpha }_{n}(t))}^{2}dt}{\mathop{\int}\nolimits_{0}^{T}dt}\right)}^{\frac{1}{2}}=\sqrt{2}{\left(\frac{1}{N}\mathop{\sum }\limits_{i = 1}^{N}{a}_{n,i}^{2}\right)}^{\frac{1}{2}}$$

**Fig. 11:**
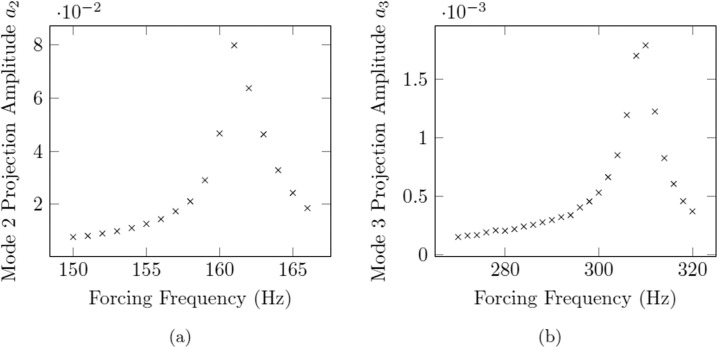
Example benchmark of results using resonance of both modes 2 and 3. **a** The resonance curve for mode 2, from which the mode 2 natural frequency is obtained. **b** The resonance curve for mode 3, from which the mode 3 natural frequency is obtained. Amplitudes *a*_*2*_ and *a*_*3*_ correspond to the mean squared projection amplitudes of modes 2 and 3, respectively. The maximum of *a*_*n*_ as a function of frequency corresponds to the natural resonant frequency of that mode. Sample is 57.345 mg Inconel 625 at 1350 °C.

The resonant frequency for a given trial is taken to be the forcing frequency which resulted in the maximum resonance of the droplet in the prescribed modal shape (mode *n* = 2 as shown in Fig. 11a or mode *n* = 3 as shown in Fig. 11b).

### Reporting summary

Further information on research design is available in the Nature Research Reporting Summary linked to this article.

## Supplementary information

Mode 2 and Mode 3 Resonance Observation in Levitated Liquid Droplet

Reporting Summary Checklist

## Data Availability

All relevant data are available from N.B.

## Code availability

All relevant codes for image analysis protocols are available from N.B.
